# A Case of Allergic Contact Dermatitis Due to Chrysanthemum After Guselkumab Therapy for Palmoplantar Pustulosis

**DOI:** 10.7759/cureus.75441

**Published:** 2024-12-10

**Authors:** Mari Kishibe, Sawa Ohtsubo, Satomi Igawa, Shinobu Matsuo, Akemi Ishida-Yamamoto

**Affiliations:** 1 Department of Dermatology, Asahikawa Medical University, Asahikawa, JPN; 2 Department of Dermatology, Matsuo Dermatological Clinic, Asahikawa, JPN

**Keywords:** allergic contact dermatitis, chrysanthemum, guselkumab, palmoplantar pustulosis, paradoxical reaction

## Abstract

Eczematous paradoxical reactions are commonly associated with anti-interleukin-17A (anti-IL-17A) antibodies. However, IL-23 p19 inhibitors can also cause similar cutaneous manifestations. We present a case of a 77-year-old Japanese woman with palmoplantar pustulosis (PPP), who developed eczematous lesions on her face, neck, and dorsum of the hands 10 weeks after initiating guselkumab treatment. Patch tests revealed a positive reaction to *Chrysanthemum* flowers and leaves, confirming a diagnosis of contact dermatitis. This was notable, as the patient had routine contact with *Chrysanthemum* every morning for years without prior allergic reactions, until after initiating anti-IL-23 p19 antibody treatment. Given the temporal association and potential causal link with guselkumab administration, the treatment was discontinued, and avoiding contact with the plant improved the contact dermatitis eruptions. Concurrently, PPP lesions remained unchanged. IL-23 inhibition is known to skew the IL-12/T-helper 1 (Th1) axis, resulting in increased interferon-γ (IFN-γ) production, a cytokine implicated in the early sensitization phase of allergic contact dermatitis (ACD). Despite prior tolerance, this case report highlights the importance of considering drug-induced ACD in patients presenting with new-onset eczema while on biologic therapy, particularly in susceptible individuals.

## Introduction

Biologic therapies, commonly referred to as biologics, comprise a class of treatments that employ monoclonal antibodies to target specific mediators within the inflammatory cascade, including cytokines. In the field of dermatology, monoclonal antibodies have become crucial therapeutic options for inflammatory skin diseases. For psoriasis, these antibodies target tumor necrosis factor-α (TNF-α), as well as interleukins such as the interleukin (IL)-12/23 p40 subunit, IL-17A, IL-17 receptor (IL-17R), and the IL-23 p19 subunit. Atopic dermatitis is similarly managed with biologics targeting IL-4 receptor α (IL-4Rα), IL-13, and IL-31. While demonstrating efficacy in treating a broad spectrum of diseases, the widespread use of biologics has revealed a potential for paradoxical reactions [1]. These reactions are characterized by the de novo development or exacerbation of pre-existing immune-mediated disorders following the initiation of treatment. The most common paradoxical reactions induced by targeted therapies with biologics are psoriasiform and eczematous eruptions. Psoriasiform paradoxical reactions are predominantly induced by TNF-α inhibitors, whereas eczematous paradoxical reactions are commonly associated with IL-17A inhibitors' administration. Eczematous paradoxical reactions typically manifest within four months following treatment initiation. The current hypothesis suggests that IL-17A inhibition skews towards T-helper 2 (Th2) by suppressing Th17. Although less common owing to the late launch, IL-23 p19 inhibitors can also cause eczematous lesions. Guselkumab is a fully human immunoglobulin-λ (IgGλ) monoclonal antibody against the p19 subunit of IL-23, extensively used for the treatment of plaque-type psoriasis and other subtypes of psoriasis, as well as palmoplantar pustulosis (PPP). Although the pathogenesis of both PPP and psoriasis is associated with the IL-23/Th17 signaling pathway, PPP exhibits a distinct immunological profile characterized by additional Th2-cell-mediated immune responses [2]. Recent clinical trial data evaluating the long-term safety profile of guselkumab in psoriatic disease have demonstrated that the incidence of adverse events necessitating treatment discontinuation was comparable to that observed in the placebo-controlled cohort [3]. However, there have been reports of patients with psoriasis developing eczematous paradoxical reactions following guselkumab administration. Notably, such reactions have not yet been reported in cases of PPP. Herein, we report the case of a patient with PPP who developed allergic contact dermatitis (ACD) to *Chrysanthemums* after receiving guselkumab treatment.

## Case presentation

**Figure 1:**
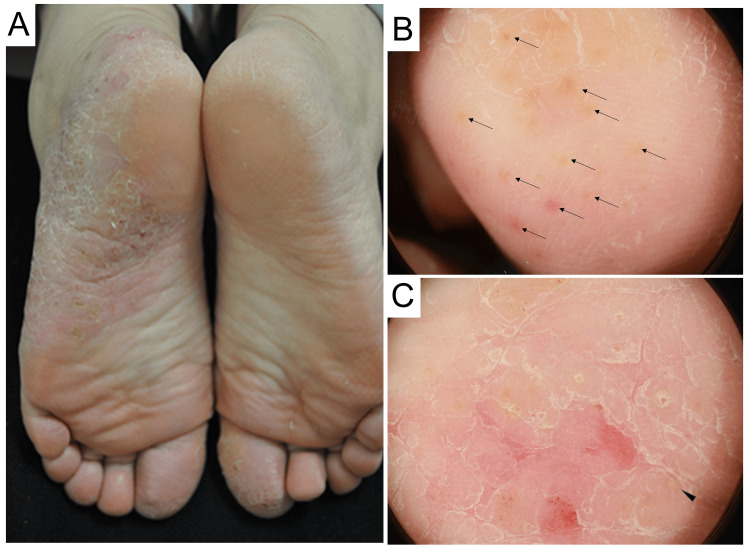
Clinical and dermoscopic findings in the plantar lesion of palmoplantar pustulosis. (A) Erythema with lamellar scales distributed on the bilateral big toes and right sole. (B-C) Dermoscopic findings revealed vesicles (arrows in B), erythema, desquamation, and pustule-vesicle (arrowhead in C).

A 77-year-old Japanese female with a 20-year history of PPP was referred to our hospital for biologic therapy, specifically guselkumab, which was the sole biological agent approved for PPP treatment in Japan at the time of presentation. The patient had previously declined other systemic therapeutic interventions. Topical steroids failed to relieve the itchy, light red erythema with lamellar scales on both her palms and soles (Figure 1A). Dermoscopic examination revealed erythema, scales, and a round yellowish area with linear vesicular surrounding, so-called pustulo-vesicle formations (Figures 1B-1C), consistent with the characteristic dermoscopic findings of PPP [4].

**Figure 2:**
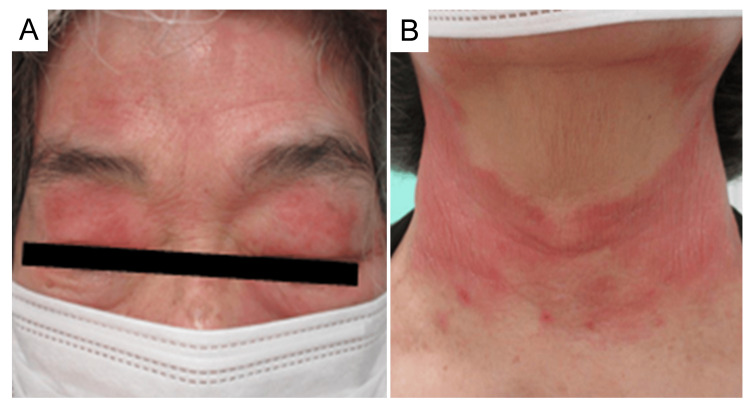
Clinical presentation of facial and cervical lesions at 10 weeks of guselkumab administration. (A) Scaly erythematous and papules on the forehead and upper eyelid. (B) Similar manifestations observed on the neck.

At the initial visit, the patient presented with a Palmoplantar Pustulosis Area Severity Index score of 4.5. Despite the relatively low score, the patient preferred biologic therapy. The patient had a history of hypertension but was not on any pharmacological treatment, and she had no history of atopic dermatitis or rhinoconjunctivitis. Baseline laboratory tests conducted prior to the initiation of biologic therapy were predominantly within normal limits, including antinuclear antibody (ANA) levels. Subcutaneous guselkumab (100 mg) was administered at weeks 0 and 4. At week 10, irregular erythema with pruritus appeared on her forehead, upper eyelids, neck, and dorsum of the hands (Figures 2A-2B).

**Figure 3:**
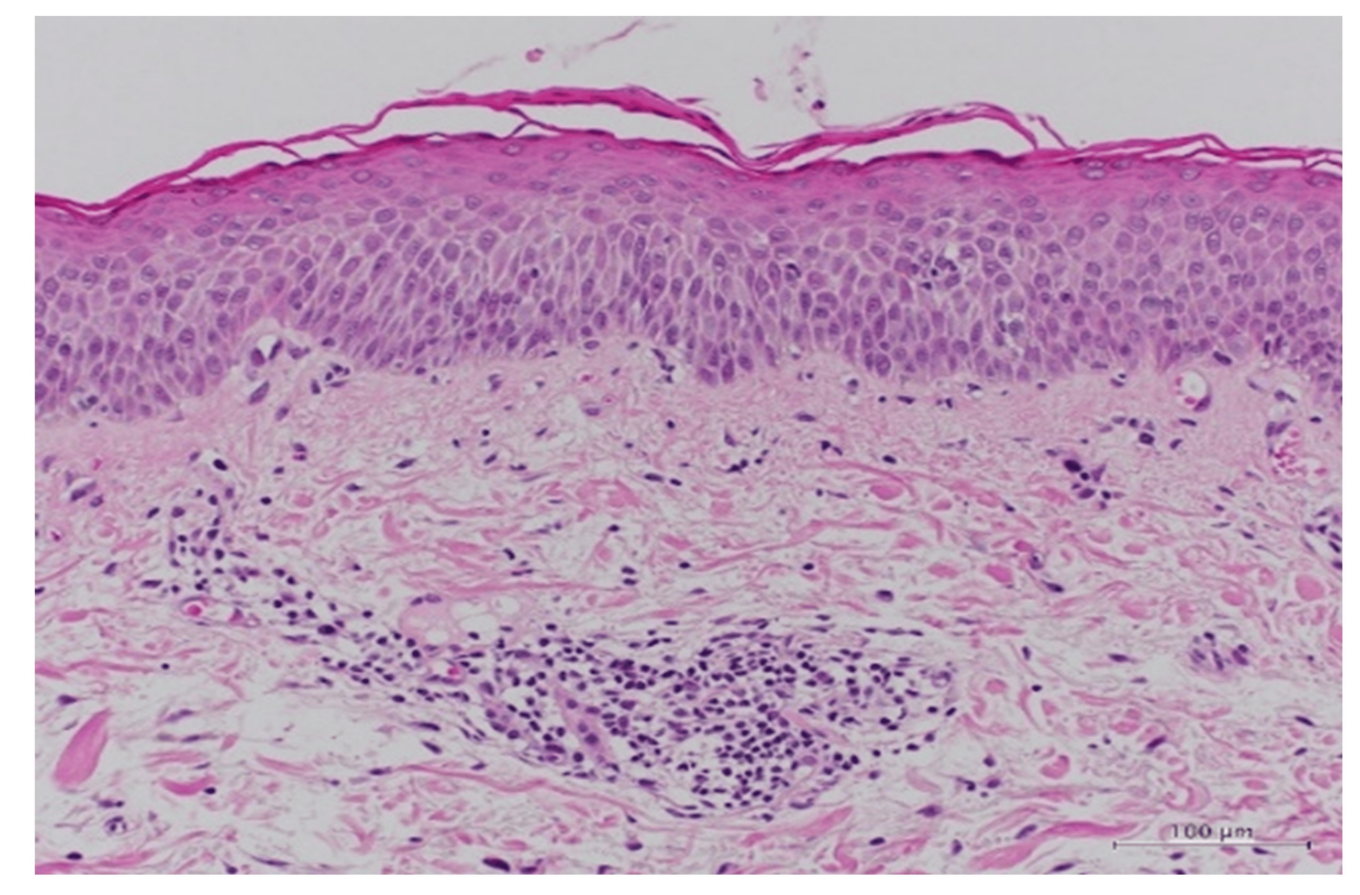
Histopathological findings of the neck lesion. Skin biopsy of the neck shows epidermal spongiosis and lymphocyte infiltration (hematoxylin-eosin; scale bars: 100 μm).

Histopathologic analysis of the neck lesions showed mild spongiosis and lymphocyte infiltrations, suggesting eczematous tissue reactions (Figure 3).

Total serum IgE levels were found to be elevated to 561.0 IU/mL (normal range: ≤232). Moreover, testing for allergen-specific IgE to *Asteraceae* plants, such as French *Chrysanthemum*, *Taraxacum*, and mugwort, showed positive results. According to the International Contact Dermatitis Research Group values, patch tests showed a positive reaction (++) to flowers and leaves of *Chrysanthemum* after 48 and 72 hours. The results of the photopatch test were inconclusive. Based on the positive patch test results, we investigated the patient’s exposure history and discovered that she had daily contact with *Chrysanthemum* every morning for years. Taken together, the patient was diagnosed with contact dermatitis to *Chrysanthemum*. Treatment was discontinued due to the temporal association and potential causal link with the guselkumab. Subsequently, the eczematous eruptions rapidly improved with the avoidance of the plant; PPP lesions did not worsen during this period.

## Discussion

Although our patient had no history of atopy and had been habitually exposed to *Chrysanthemums* for years, she had never experienced ACD until the guselkumab administration. The patient experienced this event for the first time, wherein ACD developed 10 weeks after guselkumab administration. Despite prior tolerance, this case report highlights the importance of considering drug-induced ACD in patients presenting with new-onset eczema while on biologic therapy, particularly in susceptible individuals. Eczematous paradoxical reactions are mostly associated with IL-17A inhibitors' administration, with a prevalence of 2.2-12.1% [1]. They can be considered to result from Th2/Th22 imbalance induced by IL-17A blockade; IL-22 plays a significant role in the pathogenesis of ACD. Furthermore, IL-23 p19 inhibitors have been reported to effectively improve eczematous eruptions induced by IL-17A blockers. Therefore, IL-23 p19 blockers appear to be relatively safer than IL-17A inhibitors for causing ACD or eczematous eruptions. Guselkumab, an anti-human IL-23 p19 subunit IgG1λ antibody, is the first biologic approved for the treatment of PPP [5]. Subsequently, other biologics, such as the IL-23 p19 inhibitor risankizumab and IL-17 receptor A antagonist brodalumab, have also been approved for PPP treatment. Japanese cases of PPP are more often associated with concomitant or contemporaneous focal infections than are patients with psoriasis [6]. However, IL-23 produced by human monocyte-derived dendritic cells plays a role in the early stages of PPP, similar to its role in psoriasis. In previous literature, two case reports have described the progression of eczematous lesions and ACD associated with guselkumab administration [7,8]. Both patients received guselkumab for the treatment of psoriasis. One of them, who had a history of atopic dermatitis, developed eczematous lesions 10 weeks after receiving guselkumab; the other developed ACD due to carbamates six months after treatment. Similar to the timing in previous reports, our patient developed ACD after guselkumab treatment, wherein sensitization might be more likely to be established at this time.

The possible mechanism underlying ACD due to IL-23 blockers might involve indirect inhibition of TNF-α, which could lead to the production of interferon-α (IFN-α) [7], or a skewed IL-12/Th1 axis induced by IL-23 inhibition, resulting in increased IFN-γ production [8]. This increased IFN-γ is characteristic in the early phase of ACD during sensitization. *Asteraceae* plants comprise various protein allergens and haptens, including sesquiterpene lactones, which can induce type I or IV allergies [9], including ACD, due to direct touching or airborne transmission [10]. Although *Chrysanthemums* are known to often induce ultraviolet A photosensitivity due to irradiation-induced conversion of sesquiterpene lactones into chromophores [11], in our patient, photosensitivity was excluded as a diagnosis based on inconclusive results of the photopatch test. Furthermore, the patient was not taking any medications known to induce photosensitivity, and serological testing revealed negative ANA before and after guselkumab administration. To the best of our knowledge, no previous reports of photosensitivity are associated with guselkumab. On the other hand, the temporal relationship between guselkumab therapy and the development of *Asteraceae* plant-specific IgE sensitization remains undetermined. Since baseline *Asteraceae* plant-specific IgE levels were not assessed before guselkumab administration, pre-existing elevated antibodies cannot be definitively excluded. Moreover, the potential influence of IL-23 p19 inhibition on the pathogenesis of type I hypersensitivity reactions is yet to be elucidated. Sugiura et al. reported that risankizumab, another monoclonal antibody targeting IL-23 p19, significantly elevated serum IgE levels, whereas guselkumab did not exhibit this effect [12]. This discrepancy between immunological outcomes necessitates further investigation to delineate the association between IL-23 p19 inhibition and IgE-mediated allergic responses.

Notably, patients with eczematous paradoxical eruptions have been reported to often have a personal history of atopy [1]. Recently, McCluskey et al. demonstrated a significant increase in Th17/Th2 cells in the blood and skin of patients with PPP [2], indicating that PPP might share more similarities with atopic dermatitis than with subtypes of plaque psoriasis. The prevalence of paradoxical eczematous reactions in PPP cases, compared with patients with psoriasis, remains unclear; further accumulation of clinical reports is warranted to address this question.

A limitation of this report was the inability to conduct a dermatopathologic examination of the palmoplantar lesions during the initial visit. The patient had been diagnosed with PPP based on examinations by multiple dermatologists, which aligned with our dermoscopic findings. However, given the clinical similarity between pompholyx and PPP, a careful differential diagnosis, through histopathological evaluation, should have been considered prior to the administration of guselkumab therapy.

## Conclusions

Biologic therapies for the treatment of PPP have been introduced later than those for psoriasis. Consequently, there are no case reports of eczematous paradoxical reactions induced by not only IL-17A inhibitors but also IL-23 p19 inhibitors in patients with PPP. Therefore, highlighting that anti-IL-23 p19 inhibitor therapy could induce Th1 and Th2 responses is crucial. Given the emergence of eczematous paradoxical reactions as a potential risk for susceptible individuals, particularly those with atopic profiles, it is advisable to conduct a thorough allergy history assessment. Additionally, based on our clinical experience and previous reports, we recommend careful follow-up, particularly for at least 10 weeks following therapy initiation.
